# High variability of HIV and HCV seroprevalence and risk behaviours among people who inject drugs: results from a cross-sectional study using respondent-driven sampling in eight German cities (2011–14)

**DOI:** 10.1186/s12889-016-3545-4

**Published:** 2016-09-05

**Authors:** Benjamin Wenz, Stine Nielsen, Martyna Gassowski, Claudia Santos-Hövener, Wei Cai, R. Stefan Ross, Claus-Thomas Bock, Boris-Alexander Ratsch, Claudia Kücherer, Norbert Bannert, Viviane Bremer, Osamah Hamouda, Ulrich Marcus, Ruth Zimmermann, the DRUCK Study group, Vikas Bapat, Johannes Bombeck, Kerstin Dettmer, Christiane Kerres, Andreas Hecht, Werner Heinz, Juergen Klee, Astrid Leicht, Sylke Lein, Baerbel Marrziniak, Olaf Ostermann, Norbert Scherbaum, Dirk Schäffer, Ina Stein, Lutz Wiederanders

**Affiliations:** 1 Department for Infectious Disease Epidemiology, Division for HIV/AIDS, STI and Blood-borne Infections, Robert Koch Institute, Berlin, Germany; 2 Charité University Medicine, Berlin, Germany; 3 Institute of Virology, National Reference Centre for Hepatitis C, University Hospital Essen, University of Duisburg-Essen, Essen, Germany; 4 Department of Infectious Diseases, Division for Viral Gastroenteritis and Hepatitis Pathogens and Enteroviruses, Robert Koch Institute, Berlin, Germany; 5 Department of Infectious Diseases, Division for HIV and other Retroviruses, Robert Koch Institute, Berlin, Germany

**Keywords:** PWID, Sero- and behavioural survey, HIV, Hepatitis C, Respondent-driven sampling, Second generation surveillance, Injecting drug users, Germany, Europe

## Abstract

**Background:**

People who inject drugs (PWID) are at increased risk of acquiring and transmitting HIV and Hepatitis C (HCV) due to sharing injection paraphernalia and unprotected sex. To generate seroprevalence data on HIV and HCV among PWID and related data on risk behaviour, a multicentre sero- and behavioural survey using respondent driven sampling (RDS) was conducted in eight German cities between 2011 and 2014. We also evaluated the feasibility and effectiveness of RDS for recruiting PWID in the study cities.

**Methods:**

Eligible for participation were people who had injected drugs within the last 12 months, were 16 years or older, and who consumed in one of the study cities. Participants were recruited, using low-threshold drop-in facilities as study sites. Initial seeds were selected to represent various sub-groups of people who inject drugs (PWID). Participants completed a face-to-face interview with a structured questionnaire about socio-demographics, sexual and injecting risk behaviours, as well as the utilisation of health services. Capillary blood samples were collected as dried blood spots and were anonymously tested for serological and molecular markers of HIV and HCV. The results are shown as range of proportions (min. and max. values (%)) in the respective study cities. For evaluation of the sampling method we applied criteria from the STROBE guidelines.

**Results:**

Overall, 2,077 PWID were recruited. The range of age medians was 29–41 years, 18.5–35.3 % of participants were female, and 9.2–30.6 % were foreign born. Median time span since first injection were 10–18 years. Injecting during the last 30 days was reported by 76.0–88.4 % of participants. Sharing needle/syringes (last 30 days) ranged between 4.7 and 22.3 %, while sharing unsterile paraphernalia (spoon, filter, water, last 30 days) was reported by 33.0–43.8 %. A majority of participants (72.8–85.8 %) reported incarceration at least once, and 17.8–39.8 % had injected while incarcerated. Between 30.8 and 66.2 % were currently in opioid substitution therapy. Unweighted HIV seroprevalence ranged from 0–9.1 %, HCV from 42.3–75.0 %, and HCV-RNA from 23.1–54.0 %. The implementation of RDS as a recruiting method in cooperation with low-threshold drop in facilities was well accepted by both staff and PWID. We reached our targeted sample size in seven of eight cities.

**Conclusions:**

In the recruited sample of mostly current injectors with a long duration of injecting drug use, seroprevalence for HIV and HCV varied greatly between the city samples. HCV was endemic among participants in all city samples. Our results demonstrate the necessity of intensified prevention strategies for blood-borne infections among PWID in Germany.

**Electronic supplementary material:**

The online version of this article (doi:10.1186/s12889-016-3545-4) contains supplementary material, which is available to authorized users.

## Background

According to estimations 15 million people were living with the hepatitis C virus (HCV) in the WHO European Region in 2013 [1], and 2.2 million with the human immunodeficiency virus (HIV) [2]. In most European countries people who inject drugs (PWID) are a key transmission group for blood-borne infections, including HCV and HIV [3, 4]. Studies identified several risk factors to be associated with HCV [5–11] and HIV [12, 13] infections among PWID including years of injecting, sharing of needles, syringes and other equipment, imprisonment and unprotected sex.

HIV and HCV testing are common interventions for HIV and HCV surveillance and control. They increase knowledge of HIV and HCV status, and ought to be entry points to HIV- and HCV-related treatment and care. It has been shown that opioid substitution therapy (OST) reduces injecting drug use by lowering the frequency of injecting and related unsafe practices, thereby effectively decreasing the transmission of HIV [14–16] and in combination with needle and syringes programmes (NSP) also of HCV [17]. It furthermore facilitates regular medical care and adherence to HIV and HCV treatment [18–20].

Knowledge about HIV and HCV prevalence and related behaviour amongst PWID in Germany is currently based on outdated regional studies of convenience samples. Studies providing a clear and up-to-date picture of the epidemiology of HCV and HIV amongst PWID in Germany do not exist and ongoing monitoring of infections or risk behaviours among PWID is not established. Nevertheless, regional surveys from the last decades in Germany have indicated that HCV is hyperendemic in PWID [21–24]. While the prevalence of HCV infection in the most recent population-based survey in the adult population was 0.3 %, local surveys among PWID have found prevalence ranging from 50–80 % [22–25]. High rates of infection in the PWID population were also reported from other European countries with anti-HCV prevalence ranging from 13.8 to over 90 % [26]. National estimates in Germany show that PWID are also at-risk of HIV transmission. Nearly 10 % of all estimated HIV infections were attributed to injecting drug use as of end of 2014 [27]. According to Backmund, in 2007 HIV prevalence among PWID in Germany must have been between 4.3 and 6.5 % [28] and thus, significantly higher than in the general population, where HIV infections are below 0.1 % [29]. Although there are variations across Western European countries, prevalence above 5 % among PWID has been reported in France, Spain, Ireland, Greece, Portugal, and Sweden in recent years [26]. Due to preventive efforts the number of newly diagnosed HIV infections among PWID in Germany has been declining since a peak in the late 1980ies. In 2014, an estimated 7.5 % of the 3,200 new HIV cases (240) were caused by transmission among PWID, including a sizeable proportion of approximately 25–30 % of these infections being diagnosed in Germany, but being originally acquired in Eastern or Central Europe [27]. Chronic co-infection with HCV and HIV is also common among PWID in some European countries, with a high prevalence of co-infection ranging between 15 and 70 % reported by Estonia, France, Latvia, Italy, Netherlands, Poland, Portugal and Spain [30].

To tackle the risk of blood-borne and sexually transmitted infections among PWID it is essential to combine behavioural, socio-demographic and serological data to inform the planning and implementation of effective prevention and intervention strategies [31]. By identifying knowledge gaps regarding the transmission and prevention of infections, and by revealing risky and preventive practices, factors that drive transmission among PWID can be identified and addressed. Based on such information, specific recommendations for reducing risk behaviours, scaling up prevention, treatment and care can be formulated. To obtain information on the prevalence of blood-borne infections and related behaviours for PWID in Germany, we conducted a sero-behavioural study using respondent driven sampling (RDS) in eight large cities across the country in cooperation with low-threshold drug services.

### Sampling hard-to-reach populations

Standard probability methods are generally difficult to apply in hard-to-reach populations, where a sampling frame for the targeted population is not available. RDS was introduced by Heckathorn in 1997 as a modified snowball method to recruit hard-to-reach populations [32]. Globally, more than 460 studies from 69 countries applying RDS have been published, and several studies have used RDS to recruit PWID in recent years [33]. Due to their strong social networks and because PWID often buy from and inject drugs with other PWID, RDS worked well as a recruitment method in the majority of studies [34, 35]. RDS works effectively as a sampling method, when four requirements are met [32]: first, participants need to know one another through the network of the group under study. Second, the network needs to be dense enough to attain a sample with sufficient socio-metric depth in order to reach equilibrium. The statistical rationale of RDS depends on the stabilization of the sample composition after a sufficient number of recruitment waves - the point at which the characteristic proportions remain stable, even if the recruitment continues is known, as the equilibrium [32]. The number of waves required to reach equilibrium is again linked to the third requirement: random recruitment must set in at some point to avoid that sampling is limited to a specific sub-group and only reflecting the characteristics of the seed with which the chain began. The tendency to recruit persons who are similar and thereby causing bias in the samples is termed homophily. Fourth, an enabling system to motivate participants to recruit other participants must be in place [36].

### Objectives

In this paper we present descriptive results of the first sero-behavioural study of PWID using RDS performed in Germany. The objectives are to describe basic characteristics of participants in the respective study cities focusing on i) socio-demographic factors, ii) seroprevalence of HIV and HCV, including co-infections, iii) use of health services, and iv) injecting and sexual risk behaviours. Furthermore, we assess whether RDS was effective for sampling PWID in the study cities.

## Methods

Detailed information about methodological issues has been described earlier [37].

### Overview

From 2011 to 2014, we recruited PWID using RDS across eight cities in Germany targeting a sample of 200–400 PWID in each city. All cities have a relatively large PWID community and were selected for their geographic and demographic diversity as well as the availability of low-threshold drop-in facility services. Four of the cities - Berlin, Cologne, Munich and Hamburg - have more than one million inhabitants; the four others - Essen, Leipzig, Frankfurt on the Main (Frankfurt) and Hanover- between 500,000 and 700,000.

### Study population

Eligibility for participation was defined as i) aged 16 or older, ii) self-reported injecting drug use within the past 12 months in the respective city, iii) willingness to take part in an questionnaire assisted-interview and to provide a capillary blood specimen for serological and molecular testing iv) willingness to give informed consent, and v) not having participated in the study previously.

### Sampling method

Sampling started with a small number of initial recruits (‘seeds’) in each city, selected by local partners of low threshold drug services to represent a range of characteristics (gender, country of birth, residential area and preferred low-threshold drug service, self-reported HIV serostatus, mainly preferred substance, former experience of sex work and imprisonment). All seeds were selected based on an anonymous list of PWID and their characteristics provided by the local partners. The recruitment expanded through so called ‘recruitment waves’ of peers; after the seeds recruited the first recruitment wave of participants the first recruitment wave continued to recruit the second recruitment wave of participants and so on until the targeted sample size was reached.

### Recruitment process

In each city we established between one and four RDS study sites in local low-thresholds drop-in facilities, where participants enrolled in the survey and redeemed their coupons. The recruitment coupons were valid for two weeks. Each individual received 10 EUR for participating in the study, and was paid an additional 5 EUR for each eligible drug user they recruited. To ensure anonymity and to track the recruitment process we assigned a unique numeric identifier to each participant. If a seed turned out not to be productive additional seeds were selected if needed to keep the recruitment process ongoing. Recruitment and data collection was conducted by staff of low-threshold drug services who are trained to work with PWID. This recruitment process continued until the end of the scheduled recruitment period which was reached after 7 to 9 weeks.

### Demographic, behavioural and serological data and network information

Staff of the Robert Koch Institute (RKI) conducted a two-day pre-survey training on study design, RDS methodology, standardised interviews, blood sample collection procedures and logistics for the staff of low-threshold drug services in the respective study cities. Eligible PWID had to undergo a questionnaire-assisted interview in German or Russian, wherever Russian-speaking staff was available. We asked questions regarding respondent’s demographic characteristics, their knowledge*,* attitudes*,* behaviour and practices as well as their network. Minor modifications were made in the questionnaire throughout the four years while conducting the survey. Therefore, some variables are not available for all cities. The network size was determined by asking respondents how many PWID (fulfilling the inclusion criteria for the study) they know by name who would also know the respondent by name. We also asked how many of these persons they believed they could recruit for the study. The questionnaire was based on a model questionnaire developed by the European Monitoring Centre for Drugs and Drug Addiction (EMCDDA), and additional indicators proposed by the European Centre of Disease Control (ECDC) and the Global AIDS response progress reporting (GARPR) [38–40]. Dried blood spots (DBS) on filter cards (Whatman #903) were obtained from participants’ capillary blood.. During the pilot phase of the study (cities of Essen and Berlin), DBS testing was validated in the Institute of Virology, National Reference Centre (NRZ) for Hepatitis C, at the University of Duisburg-Essen, which subsequently also performed the regular analyses on serological and molecular markers of HIV and HCV. The Division for HIV and other Retroviruses and the Division for Viral Gastroenteritis and Hepatitis Pathogens and Enteroviruses in the Department for Infectious Diseases at the RKI were in charge of the laboratory testing for the remaining six cities. The study flow and laboratory procedures including possible shortcomings arising from DBS testing are described in detail elsewhere [37, 41]. Prevalence of infection was determined by detection of anti HIV or anti HCV antibodies and detection of molecular markers for HIV and HCV by nucleic acid amplification tests. Pre- and post-test counselling were offered to participants according to international and national recommendations [42]*.*

### Measures to assess the effectiveness of RDS

For evaluation of the sampling method we applied criteria following the guidelines for “Strengthening the Reporting of Observational Studies in Epidemiology for RDS Studies” (STROBE-RDS), a checklist of essential items to present in RDS publications [33]. We provide information about the relationship of respondents with their recruiters and calculated the equilibrium and the number of recruitment waves for five key variables: I. participants’ mean age; II. proportion of male participants; III. proportion of PWID born in Germany; IV. HCV prevalence; and V. HIV prevalence. Furthermore, we describe the level of homophily among the study population. Homophily (Hx) was analysed for the following three outcomes: age, gender and HIV serology. As recommended a graphical representation of the entire recruitment network for all study cities is included. Finally, we assess whether the incentives could motivate PWID to participate in the study. Detailed material of this evaluation is attached in the Additional file.

### Statistical analysis

For data entry we used EpiData 3.0. We applied descriptive statistics by using Stata version 14.0. The crude sample proportions are presented for all cities in Tables 1, 2 and 3. The results are shown as range of proportions (min. and max. values (%)) for the respective study cities. Based on the reported network size of each participant, we used the respondent driven sampling analysis tool RDSAT version 7.1 (http://www.respondentdrivensampling.org) to define population proportions and variance estimates of each dataset [43]. We included seeds in the analysis. The number of re-samplings to determine bootstrap 95 % confidence intervals (CI) was set to 15,000 to improve the accuracy of the variance estimates and the network size outliers pulled in by 5 %. The enhanced smoothing algorithm type was employed as recommended by Johnston [44]. The RDS estimated population proportions based on the reported network are provided in the (Additional file 1: Table S1). We used RDSAT 7.1 to calculate homophily for all eight data sets. The homophily (Hx) metric is between −1 und 1. In line with Heckathorn’s suggestion we defined any value of Hx ≥ 0.3 as intermediate homophily and any value ≤ −0.3 as strong heterophily [45]. We applied Stata 14.0 to calculate equilibrium and the number of recruitment waves. Equilibrium was attained when the sample distribution from one recruitment wave to the next fell within a discrepancy of less than 2 % [46].

**Table 1:** Socio-demographic variables, 2011-14^a^

		Berlin		Essen		Leipzig		Frankfurt		Cologne		Hanover		Munich		Hamburg		range
		*n* = 337		*n* = 197		*n* = 130		*n* = 285		*n* = 322		*n* = 252		*n* = 235		*n* = 319		(min-max)
		n	%	n	%	n	%	n	%	n	%	n	%	n	%	n	%	%
Age	Mean ± SD; median; range	35.6 ± 8.8; 35.0; 18-60		37.9 ± 7.9; 38.0; 19-55		29.4 ± 7.0; 29.0; 18-55		39.6 ± 8.7; 39.0; 20-64		39.9 ± 8.4; 41.0; 18-62		39.0 ± 8.7; 39.0; 19-64		38.3 ± 8.5; 39.0; 19-63		39.8 ± 8.9; 40.0; 17-65		29-41
	<25 years	30	9.0	9	4.6	35	26.9	6	2.1	11	3.4	15	6.0	16	6.8	13	4.1	2.1-26.9
Gender	Female	62	18.5	39	19.8	29	22.3	73	25.8	73	22.7	50	19.8	83	35.3	71	22.3	18.5-35.3
Country of birth	Foreign-born	103	30.6	38	19.3	12	9.2	59	20.7	67	20.8	57	22.6	39	16.6	84	26.3	9.2-30.6
	Eastern Europe and Former Soviet Union^b^	83	24.7	20	10.2	8	6.3	34	11.9	22	6.8	40	15.9	23	9.8	62	19.5	6.3-24.7
Educational level	No school certificate	52	15.4	38	19.4	17	13.2	24	8.5	67	20.8	34	13.5	20	8.5	50	15.8	8.5-20.8
	Completed lower secondary - 9th grade	143	42.4	109	55.6	62	48.1	147	52.1	130	40.4	119	47.2	137	58.3	135	42.6	40.4-58.3
	Completed 10th grade	121	35.9	38	19.4	45	34.9	78	27.7	74	23.0	78	31.0	52	22.1	95	30.0	19.4-35.9
	High school graduate	21	6.2	11	5.6	5	3.9	33	11.7	51	15.8	21	8.3	26	11.1	37	11.7	3.9-15.8
Main source of income in the past 12 months	Regular job/Unemployment benefit	56	16.8	29	14.7	25	19.4	67	23.8	59	18.3	61	24.2	67	28.6	84	26.8	14.7-28.6
	Social benefits/pension	289	86.5	176	89.3	111	86.1	231	81.9	291	90.4	217	86.1	192	82.1	229	72.9	72.9-90.4
Homelessness	In the last 12 months^c^	29	8.6	28	14.2	28	21.5	79	28.7	48	15.3	17	6.8	27	11.5	55	17.3	6.8-28.7
	Ever	216	64.5	128	65.0	100	76.9	210	73.9	218	68.1	133	52.8	139	59.2	225	70.8	52.8-76.9

^a^Footnote (Table 1): Because not all participants replied to every variable, some variables include missing values. This means that the city-specific denominator for some variables might be lower than the n displayed at the top of the table

^b^Eastern Europe and Former Soviet Union: Includes PWID reporting being born in the following 24 countries: Azerbaijan, Bosnia & Herzegovina, Bulgaria, Croatia, Czech Republic, Estonia, Georgia, Hungary, Kazakhstan, Kosovo, Kyrgyzstan, Latvia, Lithuania, Montenegro, Poland, Romania, Russian Federation, Serbia, Slovakia, Slovenia, Tajikistan, Ukraine, Uzbekistan and Yugoslavia

^c^Main reported form of residence, includes living on the street and in homeless shelters

**Table 2:** Serological and molecular findings for HIV and HCV and use of health care services, 2011-14^a^

	Berlin		Essen		Leipzig		Frankfurt		Cologne		Hanover		Munich		Hamburg		range
	*n* = 337		*n* = 197		*n* = 130		*n* = 285		*n* = 322		*n* = 252		*n* = 235		*n* = 319		(min-max)
Serological and molecular findings	n	%	n	%	n	%	n	%	n	%	n	%	n	%	n	%	%
HIV +	13	3.9	12	6.1	0	0	26	9.1	5	1.6	22	8.7	7	3.0	16	5.0	0.0-9.1
HCV seroprevalence; Anti-HCV + and/or HCV-RNA +	185	54.9	143	72.6	55	42.3	189	66.3	229	71.1	189	75.0	149	63.4	222	69.6	42.3-75.0
Cleared infection; Anti HCV+, HCV RNA -	60	17.8	54	27.4	25	19.2	46	16.1	76	23.6	53	21.0	64	27.2	79	24.8	16.1-27.2
Chronic infection; Anti HCV+, HCV RNA +	125	37.1	89	45.2	30	23.1	143	50.2	153	47.5	136	54.0	85	36.2	143	44.8	23.1-54.0
Seroconverters; Anti HCV-, HCV RNA +	4	1.2	3	1.5	7	5.4	5	1.8	15	4.7	5	2.0	2	0.9	6	1.9	0.9-5.4
Co-infections: Anti HIV+, Anti HCV + and/or HCV RNA+	12	92.3	12	100.0	-	-	21	80.8	3	60.0	19	86.4	6	85.7	11	68.8	60.0-100.0
Use of health care services and testing history																	
Use of harm reduction service (last 30d)	^b^	-	^b^	-	^b^	-	256	90.5	261	81.3	221	87.7	185	78.7	284	89.0	78.7-90.5
Currently in OST	135	40.3	85	43.2	40	30.8	129	45.3	213	66.2	109	43.3	129	55.1	179	56.3	30.8-66.2
Ever receiving OST	244	72.8	170	86.3	71	54.6	233	81.8	279	86.7	211	83.7	208	88.9	254	79.6	54.6-88.9
Ever tested for HIV	298	90.6	184	94.4	98	76.6	277	97.5	302	95.0	233	94.3	220	96.1	301	95.0	76.6-97.5
Tested for HIV (last 12 m)^c^	173	57.1	125	68.3	54	43.9	171	66.3	207	69.7	137	60.6	146	69.9	197	68.4	43.9-69.9
Ever tested for HCV	287	89.4	184	94.9	85	70.3	264	94.6	290	93.6	224	91.8	215	96.0	269	90.0	70.3-96.0
Tested for HCV (last 12 m)^d^	70	49.0	39	59.1	21	28.8	44	54.3	60	58.8	30	42.3	52	75.4	47	46.5	28.8-75.4

^a^Footnote (Table 2): Because not all participants replied to every variable, some variables include missing values. This means that the city-specific denominator for some variables might be lower than the n displayed at the top of the table

^b^data not collected

^c^Excluding those with a diagnosis older than 12 months

^d^Denominator includes those never tested, those never tested positive and those who had their first HCV diagnosis in the last 12 months

**Table 3:** Substance use, sharing behaviours, sexual risks and incarceration experience, 2011-14^b^

	Berlin *n* = 337		Essen *n* = 197		Leipzig *n* = 130		Frankfurt *n* = 285		Cologne *n* = 322		Hanover *n* = 252		Munich *n* = 235		Hamburg *n* = 319		range (min-max)	
	n	%	n	%	n	%	n	%	n	%	n	%	n	%	n	%		%
Years of injecting (mean ± SD; median; range)	13.4 ± 8.8; 13.0; 0-43		16.9 ± 9.0; 17.5; 0-40		10.0 ± 6.1; 10.0; 0-30		17.4 ± 10.0; 16.0; 0-44		17.9 ± 9.0; 18.0; 0-42		17.6 ± 9.4; 18.0; 0-43		17.4 ± 9.1; 17.5; 0-45		18.0 ± 9.4; 18.0; 0-43		10.0-18.0	
Injecting <2 years	26	7.8	10	5.1	14	11.1	14	4.9	13	4.0	8	3.2	12	5.2	19	6.0	3.2-11.1	
Injected drugs (last 30d)	279	82.8	170	86.3	99	76.2	238	83.5	263	81.7	202	80.2	187	79.6	282	88.4	76.2-88.4	
Injected daily (last 30d)	108	39.1	57	33.7	29	30.2	72	31.2	84	32.3	65	32.2	32	17.2	69	24.7	17.2-39.1	
Heroin consumed (last 30d)	280	83.1	154	78.2	89	68.5	224	78.6	275	85.4	189	75.0	133	56.8	201	63.2	56.6-85.4	
Cocaine consumed (last 30d)	125	37.1	120	60.9	23	17.7	125	44.0	150	46.7	166	65.9	49	20.9	255	79.9	17.7-79.9	
Crack consumed (last 30d)	8	2.4	6	3.1	1	0.8	204	71.6	6	1.9	147	58.6	1	0.4	146	45.9	0.4-71.6	
Methamphetamine (last 30d)	9	2.7	0	0.0	87	67.4	4	1.4	3	0.9	0	0.0	15	6.4	7	2.2	0.0-67.4	
Unsafe Use behaviour																		
Shared needle/syringes (last 30d)	40	15.0	32	19.2	17	17.9	11	4.7	51	19.5	44	22.3	24	13.2	29	10.6	4.7-22.3	
Shared equipment (spoon, filter, water) (last 30d)	99	37.5	56	33.5	40	42.6	99	43.8	84	33.6	64	33.0	61	34.5	87	33.0	33.0-43.8	
Sexual risks																		
Sexual intercourse (last 12 m)	237	72.9	144	77.0	110	84.6	228	80.9	224	74.2	182	73.7	193	82.1	237	75.0	72.9-84.6	
No condom use during last sexual intercourse	130	56.1	59	44.9	45	63.4	125	56.3	138	61.6	109	60.2	130	69.1	134	56.5	44.9-69.1	
Last sex partner was IDU	134	57.5	73	51.4	70	68.6	139	65.6	120	56.1	105	64.4	127	69.4	118	54.1	51.4-69.4	
Incarceration																		
Ever incarcerated^a^	257	76.5	169	85.8	108	83.1	239	84.2	262	81.9	214	85.6	171	72.8	254	79.6	72.8-85.8	
Total duration of incarceration (yr) Mean ± SD; median; range	3.8 ± 4.5; 2.0; 0-26		5.3 ± 5.2; 4.0; 0-23		3.4 ± 4.3; 2.0; 0-24		5.1 ± 5.5; 3.0; 0-29		4.8 ± 5.0; 3.0; 0-23		6.5 ± 6.3; 5.0; 0-30		3.2 ± 3.6; 2.0; 0-15		5.0 ± 4.9; 4.0; 0-20		2.0-5.0	
Injecting in prison (ever)	101	39.3	55	32.7	19	17.8	59	24.7	78	29.9	78	37.0	35	20.5	70	27.7	17.8-39.3	
Shared needle/syringes/equipment (among those injecting during their last imprisonment)	32	33.0	20	37.7	7	38.9	20	36.4	38	49.4	32	41.0	14	40.0	33	48.5	33.0-49.4	
Unprofessionally tattooed/pierced in prison (ever)	81	24.2	67	34.5	39	32.2	79	27.8	97	30.2	76	30.3	45	19.4	71	22.3	19.4-34.5	

^a^Including juvenile arrest/prison, pre-trial custody, prison, forensic commitment

^b^Footnote (for Table 3): Because not all participants replied to every variable, some variables include missing values. This means that the city-specific denominator for some variables might be lower than the n displayed at the top of the table

## Results

### Socio-demographic characteristics of participants

Overall, we recruited a total of 2,079 participants in the multicentre survey, of which two did not meet our eligibility criteria. Most of the interviews took around 45 min to 1 h to complete. In each city except Leipzig (*n* = 130) a sample size between 200–400 PWID was achieved (see Table 4). In all cities the proportion of female participants ranged between 18.5 and 35.3 %. The median age of participants varied between 35–41 years – except in Leipzig where the median age was 29 years. Accordingly, the proportion of PWID younger than 25 years was higher in Leipzig (26.9 %) compared to the remaining seven cities (2.1–9.0 %). Leipzig was also an exception with regards to country of origin of the participants. Foreign-born participants accounted for 9.2 % in Leipzig and ranged between 16.6 and 30.6 % in the other seven cities. The proportion of participants born in Eastern Europe and Former Soviet Union ranged between 6.3 in Leipzig and 24.7 % in Berlin.

**Table 4:** Details of the recruitment procedures using RDS in eight German cities, 2011-14

	Berlin	Essen	Leipzig	Frankfurt	Cologne	Hanover	Munich	Hamburg
	(*n* = 337)	(*n* = 197)	(*n* = 130)	(*n* = 285)	(*n* = 322)	(*n* = 252)	(*n* = 235)	(*n* = 319)
Target sample size	300-350	200	200	300	300	200-250	200	300
Month and year of recruitment	05-07. 2011	10-12. 2011	10-12.2011	01-03.2013	04-05.2013	07-09.2013	10-12.2013	03-05.2014
Time of recruitment	8 weeks	8 weeks	7 weeks	9 weeks	8 weeks	8 weeks	8 weeks	8 weeks
No. of study sites	4	1	2	2	1	1	1	1
No. of seeds in total	19	18	13	11	12	7	13	9
No. of unproductive seeds	4	6	4	2	0	4	3	0
Max. number of recruitment waves	13	10	8	20	13	14	14	20
Coupon received from partner	^a^	^a^	7 %	5 %	2 %	4 %	3 %	2 %
Coupon received from acquaintance	^a^	^a^	78 %	64 %	84 %	50 %	67 %	65 %
Coupon received from stranger	^a^	^a^	15 %	31 %	14 %	46 %	30 %	33 %

^a^data on the relationship to the recruiter was not collected in first two study cities

The majority of participants in all cities had completed lower secondary school (40.4–58.3 %) and between 8.5 and 20.8 % had not completed any school. Between 72.3–90.4 % reported currently receiving social benefits/pensions. Furthermore, more than half of the participants in all cities had been homeless at least once in life (52.8–76.9 %). Between 6.8 (in Hanover) and 28.7 % (in Frankfurt) of the participants reported being homeless or staying in homeless shelters as their main residence in the past 12 months (Table 1).

### Seroprevalence of HIV and HCV and use of health care services

**Fig. 1:**
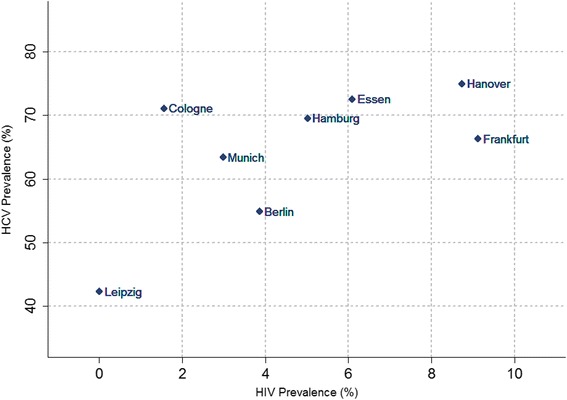
HIV and HCV seroprevalence in the eight cities (%)

HIV prevalence amongst participants varied between 0 % in Leipzig and 9.1 % in Frankfurt. HCV prevalence (Anti-HCV or HCV-RNA positive or both) ranged from 42.3 in Leipzig to 75.0 % in Hanover (Fig. 1), while HCV viremic infections (HCV-RNA positive) were found to range from 23.1 in Leipzig to 54.0 % in Hanover. HCV-RNA in the absence of anti-HCV antibodies was detected in 0.9 % of the cases in Munich and in 5.4 % of the cases in Leipzig, indicating recent HCV infections before seroconversion. HCV co-infections amongst the HIV positive participants were detected between 60.0 % of cases in Cologne and 100 % in Essen.

### Use of health services and testing history

Data on utilisation of low-threshold drug services (in the last 30 days) was collected in five of the eight study cities. The proportion of PWID who visited a low-threshold drug service in the last 30 days ranged from 78.7 to 90.5 %. About three out of five participants in each of the eight cities (54.6–88.9 %) reported ever receiving opioid substitution therapy (OST). Currently receiving OST varied between 30.8 in Leipzig and 66.2 % in Cologne. In all cities the vast majority had been tested for HIV during their lifetime (76.6–97.5 %). Undergoing an HIV test in the last 12 month was reported by 43.9 % (Leipzig) to 69.9 % (Munich). The majority of the study population in each city reported ever being tested for HCV antibodies (70.3–96.0 %), while having been tested recently (12-month prevalence) was reported between 28.8 (Leipzig) and 75.4 % (Munich) (Table 2).

### Recent substance use and risk behaviours

The median number of years since first injection was 10 years in Leipzig, 13 years in Berlin and between 16 and 18 years in the remaining cities. In seven cities, the proportion of participants who initiated injecting in the last two years ranged from 3.2 in Hanover to 7.8 % in Berlin. In Leipzig one out of ten (11.1 %) had started injecting in the last two years.

Injecting drugs in the last 30 days was reported by more than three out of four participants in all eight cities (76.0–88.4 %) and daily injection in the last 30 days varied between 17.2 in Munich and 39.1 % in Berlin. In the last 30 days Heroin was the most frequently used substance (all routes of administration) in five cities (Berlin: 83.1 %; Essen: 78.2 %; Cologne: 85.4 %; Hanover: 75.0 % and Munich 56.8 %); while in Leipzig methamphetamine was the most frequently reported substance (67.4 %). In the other seven cities methamphetamine was less common (0.0–6.4 %). Crack was used by a high proportion of participants in three cities (Frankfurt: 71.6 %; Hanover: 58.6 % and Hamburg: 45.9 %) while reported by much lower proportions in the remaining five cities (0.4–3.1 %). Cocaine use was lower in Berlin (37.1 %), Leipzig (17.7 %) and Munich (20.9 %) compared to Frankfurt (44.0 %), Cologne (46.7 %), Essen (60.9 %), Hanover (65.9 %) and Hamburg (79.9 %). Sharing of unsterile needles and syringes (n/s) in the last 30 days was reported by 10.6 % in Hamburg and up to 22.3 % in Hannover among participants who reported having injected during the last 30 days.; only in Frankfurt the proportion was lower (4.7 %). Recent sharing of unsterile equipment like spoons, filters or water for injection with other injectors was reported by 33.0 % in Hanover and Hamburg and by up to 43.8 % in Frankfurt among persons who injected during the last 30 days.

### Sexual risk behaviours

Between 72.9 and 84.6 % of participants reported engaging in sexual intercourse in the last 12 months, of which 44.9 % in Essen and up to 69.1 % in Munich reported not having used a condom at last sexual intercourse. More than half of the participants reported that their last sexual partner was also injecting drugs (51.4 % in Essen and up to 69.4 % in Munich).

### History of incarceration

Imprisonment (ever) was reported by the majority of participants in all cities (72.8–85.8 %) and median of total duration of incarceration ranged between 2.0 years in Leipzig, Berlin and Munich to 5.0 years in Hanover. Of those who had ever been in prison, between 17.8 % in Leipzig and 39.3 % in Berlin reported injecting drugs while incarcerated. Of these, between 33.0 and 49.4 % had shared n/s or other equipment when injecting during their last incarceration. Tattooing and piercing during imprisonment were reported by 19.4 % of the participants in Munich and up to 34.5 % of the participants in Essen.

The detailed data on substance use and risk behaviours in the last 30 days are shown in Table 3.

### Evaluation of the sampling method: respondent driven sampling

Between 7 and 19 seeds started the recruitment process in the respective cities. The sample of Leipzig (*n* = 130) reached a maximum of eight recruitment waves, while the samples of Frankfurt and Hamburg reached a maximum of 20 recruitment waves (Table 4). Equilibrium and homophily were assessed after the recruitment process had been completed. We reached equilibrium for four of the following five key variables: I. participants’ median age; II. proportion of male participants; III. proportion of PWID born in Germany; IV. HCV prevalence; and V. HIV prevalence in all study cities except in Leipzig. Equilibrium for HIV prevalence was not attained in our sample of Frankfurt, Hanover and Cologne. The results of these analyses are presented in the (See Additional file 2: Figure S1a, Additional file 3: Figure S1b, Additional file 4: Figure S1c, Additional file 5: Figure S1d, Additional file 6: Figure S1e and Additional file 7: Figure S2). Respondents and recruiters had the following relationships: Most participants (54–86 %) received their coupons from their partner or from an acquaintance. Between 14 % of the participants in Cologne and up to 46 % of the participants in Hanover received their coupons from a stranger (Table 4). The reported network size defined as “how many people who injected drugs in the last 12 months do you know (and they know you)” ranged from 0–1400 individuals. We did observe random recruitment among the participants. In Frankfurt, Cologne and Hamburg young participants (<25 years) demonstrated a strong negative homophily, indicating that younger participants only recruited older participants (Hx = −1). Among the female participants, only women in Leipzig demonstrated a negative homophily (Hx = −0.37). In Cologne and Munich HIV positive participants only recruited HIV negative participants (Hx = −1) while HIV negative participants in Cologne demonstrated intermediate homophily recruiting mostly other HIV negative participants (Hx = 0.67). The recruitment chains in Cologne and Hamburg show a very late recruitment of HIV positive PWID in the study sample (See Additional file 8: Figure S4e and Additional file 9: Figure S4h)**.** In those two city samples the recruitment chains have often ended once HIV positive participants were recruited. A graphical representation of the recruitment networks (including HIV and HCV serostatus) in each study city is displayed in the (See Additional file 10: Figure S4a-b, Additional file 11: Figure S4c-d, Additional file 8: Figure S4e-f, Additional file 9: Figure S4g-h).

In all cities we observed a decreased interest in participation in the days following the monthly “social benefit”-payment. We did not experience recruitment challenges such as commercial exchange of coupons, imposters or duplicate recruits.

## Discussion

This paper presents first findings of the first large bio-behavioural survey among PWID using RDS in Germany. With a study sample of 2,077 participants, the results of the study provide recent data on current HCV and HIV prevalence, socio-demographical factors and behaviours among PWID in Germany. Our results show that HCV is endemic among the study populations (42.3–75.0 %). This result is similar to estimations from available regional surveys and reported data from several European countries [47]. Viremic HCV infections among the participants were found to range between 23.1–54.0 %. In contrast to previous findings from sub-regional surveys in Germany [28], HIV prevalence varied widely between the city samples ranging from 0 % in Leipzig to 9.1 % in Frankfurt. HIV prevalence of more than 5 % was found in four of the city samples. These results are higher compared to reported data on the HIV prevalence in PWID in many other Western European countries, such as the United Kingdom, Denmark, Norway, Austria, or Luxemburg, but still lower than in countries like Italy, Portugal and France [48].

HIV and HCV seroprevalences were both found to be geographically heterogeneous. While Leipzig was the city sample with the lowest prevalences, participants in Essen, Hanover and Frankfurt had high levels of HIV and HCV infections. The differences between the locations might be due to several factors. We identified three characteristics that might be associated with the different levels of HIV and HCV prevalence across the cities: First, age (closely linked with duration of injecting), second, drug use patterns in each city and third, the history of intravenous drug use and the HIV epidemic in the region. In Essen, Hanover and Frankfurt (all city samples with high levels of HIV and HCV infections) study participants were generally older and duration of injecting was longer than in Leipzig. This is consistent with the trend of aging injecting drug user-populations in Germany and Europe at large. The longer a person has injected drugs, the more likely it is that this person will have been exposed to blood-borne pathogens [5]. The sample of Cologne seems to be an exception with an unexpected low HIV seroprevalence. However, as described, in this city HIV-positive persons were recruited only in a late stage of the recruitment process shortly before the end of the study. We therefore might underestimate the true HIV prevalence in this city sample. Further research will be needed to explain this discrepancy.

The different HCV and HIV prevalence might also be associated with the varying use of cocaine, crack and methamphetamines in the cities. Cocaine was found to be most common in Hamburg, Hanover and Essen while crack was mostly used in Frankfurt, Hanover and Hamburg, all of which are cities with a high HIV and HCV prevalence. Cocaine and crack have a shorter biological half-life and need to be consumed more frequently than other substances in order to maintain their effect [49, 50]. PWID who use cocaine or crack may consequently be more exposed to unsafe use than PWID who use substances requiring less injections. Methamphetamine was found to be most common in Leipzig. This confirms an increasing trend of methamphetamine use in border regions to Czech Republic like Saxony reported in the last years [51–54]. Methamphetamine has a longer biological half-life than heroin and may thus be less frequently injected [55]. Furthermore, the distinct demographic characteristics and consumption patterns in Leipzig might be related to the division of Germany to East and West until 1990. A drug scene in the former East probably developed only after the German re-unification in 1990. While HIV incidence among PWID in West Germany peaked in the mid-1980s, in the Eastern part of Germany the spread of HIV was delayed in time [56]. The delayed epidemic of intravenous drug use and associated blood-borne infections in East Germany are reflected in our results. According to the national HIV case-reporting system HIV is present among PWID in Leipzig with two reported cases in 2012 and three in 2015 [57]. This indicates an ongoing HIV transmission, albeit on a low level [58]. In our sample of Leipzig, we found the highest proportion of new injectors and the highest proportion of participants testing HCV RNA-positive in the stage of seroconversion, likewise indicating ongoing transmissions. This result is consistent with evidence that HCV incidence is rapidly increasing among new injectors [7, 59].

### Injecting and sexual risk behaviours

Risk behaviours (30-day prevalence) like using unsterile paraphernalia (spoons, filters or water), sharing of unsterile n/s and practicing unsafe sex, also with non-IDU (12-month prevalence), was reported in all city samples. In the last two decades, harm reduction interventions like NSP in high income countries, including Germany have led to a remarkable decline in the re-use of unsterile n/s and in HIV incidence among PWID [60–63]. From our findings we must conclude that either the access to clean n/s is still insufficient and/or that there are still knowledge gaps around transmission and preventive behaviour of HIV and HCV. In other studies, sharing of other paraphernalia still persists at higher levels than sharing n/s among PWID [64–66]. This was also reflected in our study: sharing other paraphernalia appeared much more common than sharing n/s which was most prevalent in the sample of Frankfurt. In this particular case, the high discrepancy might be related to the high number of crack users. Crack is generally linked to a high consumption frequency, and thus with an increased use of paraphernalia.

Several studies have demonstrated that HCV is more infectious than HIV and a prolonged survival of HCV in syringes and non-syringe injecting paraphernalia has been shown [67, 68]. Not surprisingly and in line with data from other Western European countries, HCV infections therefore appeared to be more prevalent within the study population than HIV [26].

High proportions of the participants reported that their last sexual intercourse was unprotected. The last sexual partner was frequently reported to be an injecting drug user as well, but it was also not uncommon that the sexual partner was a non-PWID. The reported sexual behaviours thus demonstrate the potential risk of spreading HIV through the sexual route. While other studies have demonstrated the higher risk of non-PWID to acquire HIV [69], in Germany, little is known about HIV prevalence among the non-injecting sexual partners of PWID and their potential risk of being a bridge population between PWID and the general population. Therefore further research is needed to better understand the HIV/HCV prevention needs of sex partners of PWID who do not inject drugs themselves.

The study found high rates of incarceration (at least once in lifetime) among the study participants. Unsafe drug use and tattooing/piercing in prison were reported as common practices while in prison, thus constituting important risk factors for the transmission of HIV and HCV. Several studies have shown that not only drug use but also HIV and HCV infections among people in prison are of major concerns in Germany [70, 71]. The provision of harm reduction services in the criminal justice system seems to be insufficient, only one of 186 prisons in Germany offers NSP [72], and there are large variations regarding the availability of OST in prisons across the federal states [70, 73].

Our study shows that HCV and HIV testing rates (12-month prevalence) remained moderate to high in the study populations in comparison to other risk groups, like men who have sex with men [74]. Especially the large variation in the HCV testing rates across the study cities may be linked to the variation of participants who reported undergoing OST at the time of participating, but this needs further investigation.

An important limitation of our study is that it only provides a snap shot of the HIV and HCV epidemiology among PWID in Germany but it does not allow determination of cause-effect relationships. Furthermore, the selection of our study cities was based on the availability of low-threshold drug services in the cities willing and able to participate in the study. Since national representative data on PWID in Germany are not available, we cannot claim that the PWID recruited in the chosen cities are representative for all PWID in Germany.

### Respondent driven sampling

The application of RDS as a method to recruit PWID was successful. We reached the targeted sample size within the set time frames in all cities except in one, and our primary and secondary incentives seemed appropriate to motivate PWID to enroll in the study. This is in line with other studies, showing that RDS is an effective recruitment method among PWID [35, 75].

The choice of low-threshold drug services as study venues probably increased the willingness of participation. We observed long recruitment chains in seven of the eight city samples, indicating that PWID are well connected via social networks or through making use of the low-threshold drug services. However, we cannot exclude selection bias of the city samples due to oversampling of persons as initial seeds as well as participants who showed communicative competence, well understood the study flow and the background of the study, and who were willing to recruit others for participation. Persons with a lower bonding to the drug scene or less communicative skills might be underrepresented in the samples.

Due to the extensive questionnaire, we refrained from asking the participants how many PWID rejected their coupons during the recruitment process. Reasons for rejecting and the number of PWID who refused are therefore unknown. It is possible that unknown barriers restricted participation and potentially created a bias. Yet we assume that our samples mostly attained adequate socio-metric depth, given that equilibrium was reached for four out of five key variables while reaching up to 20 waves in our samples. However, not all city samples allow robust weighting of results, as equilibrium was not reached in all and the length of the recruitment chains was too short in the city of Leipzig. In this city we recruited PWID in two low-threshold drug services with alternating study operating hours. This seems to have confused some study participants and it is possible that further potential participants were lost due to this fact. Equilibrium could be reached in the other seven samples, showing that bias introduced by the initial non-randomly selected seeds could be eliminated in these samples.

Despite the popularity and the widely applied methodology of RDS as a sampling method it is not known whether RDS can generate unbiased estimates. The assessment of RDS as a method of data analysis (RDS inference) is challenging as it often fails to produce precise results due to the unknown underlying truth [76]. Also, the key variable used to generate the RDS-generated estimates is the reported network size of the participant which may not have been consistently addressed by all interviewers or not consistently understood by all participants, leading to a large range and thereby further uncertainty about the validity of the RDS estimates. In 2012 McCreesh et al. have performed a RDS study with known characteristics in order to assess the precision and relevance of RDS inference and found that RDS failed to reduce bias when it occurred, and even tended to overestimate biased adjusted results [77]. In case biases occur in practice the method is not designed to correct for the sources of biases. RDS-generated estimates should therefore be interpreted with caution and are only shown in the Additional file 1: Table S1.

## Conclusions

To best of our knowledge this study is the first bio-behavioural study using RDS in Germany successfully recruiting members of the target population. This paper presents basic descriptive results for key variables in all of the eight study cities. HCV was found to be hyperendemic within the study population. HIV and HCV seroprevalence were geographically heterogeneous, although unsafe use behaviour, such as sharing n/s and other paraphernalia, unsafe sex, and incarceration was common among all city samples.

Based on our findings, efforts to reduce sharing of non-syringe paraphernalia and to further reduce the use of unsterile n/s are urgently needed in Germany. We furthermore recommend to scale up and increase the access to multilevel and combined HCV and HIV prevention, including antiviral treatment, OST and voluntary counselling and testing (VCT) for PWID. Our study suggests that there might be opportunities to better integrate VCT services in low-threshold drug services, as they were used by up to 90 % of the participants (30-day prevalence). Based on the large regional differences observed in our study, we suggest developing context specific interventions. Harm reduction programmes should particularly consider new injectors. Internationally, there is consensus in the scientific discourse about the need to provide prevention, treatment and care interventions for all, people living in freedom as well as for prisoners [78].

Further in-depth analyses of the collected data will reveal possible associations between infections and behavioural factors and other characteristics, to derive concrete recommendations for current prevention strategies for HCV and HIV among PWID.

## Additional files

Additional file 1: Sample proportion and estimated population proportion estimates for all variables. (XLS 75 kb) Additional file 2: HCV prevalence. (TIF 566 kb) Additional file 3: HIV prevalence in all study cities except in Leipzig. (TIF 518 kb) Additional file 4: Proportion of male participants. (TIF 535 kb) Additional file 5: Proportion of participants born in Germany. (TIF 628 kb) Additional file 6: Participants´ median age. (TIF 494 kb) Additional file 7: Number of recruits per recruitment wave. (TIF 695 kb) Additional file 8: Sample of Cologne (2013); n=322 (12 seeds) and sample of Hanover (2013); n=252 (7 seeds). (TIF 3197 kb) Additional file 9: Sample of Munich (2013); n=235 (13 seeds) and sample of Hamburg (2014); n=319 (9 seeds). (TIF 3828 kb) Additional file 10: Sample of Berlin (2011); n=337 (19 seeds) and sample of Essen (2011); n=197 (19 seeds). (TIF 2489 kb) Additional file 11: Sample of Leipzig (2012); n=130 (13 seeds) and sample of Frankfurt (2013); n=285 (11 seeds). (TIF 2504 kb) Additional file 12: Homophily (Hx) in eight city samples (age, gender, HIV seroprevalence). Cities: Berlin (B), Essen (E); Leipzig (L); Frankfurt (F); Cologne (C); Hanover (H); Munich (M); Hamburg (HH). The homophily Hx shows the tendency of individuals in a group having social bonds with other individuals similar to them. Hx = 0 means that the formation of social bonds is independent of group membership. Hx=1 mean no social bonds to outsiders exist. Hx= -1 means all social bonds are formed with people outside the group [44]. (TIF 690 kb)

## Abbreviations

CI: Confidence intervals
DRUCK-study: Drugs and chronic infectious diseases study (Studie zu “Drogen und chronischen Infektionskrankheiten”)
ECDC: European centre of disease prevention and control
HCV: Hepatitis C virus
HCV RNA: Hepatitis C virus ribonucleic acid
HIV: Human immunodeficiency virus
IDU: Injecting drug users
NSP: Needle and syringe exchange programme
n/s: Needles and syringes
OST: Opioid substitution treatment
PWID: People who inject drugs
RKI: Robert Koch-Institute
UNODC: United Nations Office on Drugs and Crime
RDS: Respondent-driven sampling
VCT: Voluntary testing and counselling
