# Supplementary figures and images for: High variability of HIV and HCV seroprevalence and risk behaviours among people who inject drugs: results from a cross-sectional study using respondent-driven sampling in eight German cities (2011–14)
Source: BMC Public Health. 2016 Sep 5;16(1):927. doi: 10.1186/s12889-016-3545-4 (PMC5011883; doi:10.1186/s12889-016-3545-4)

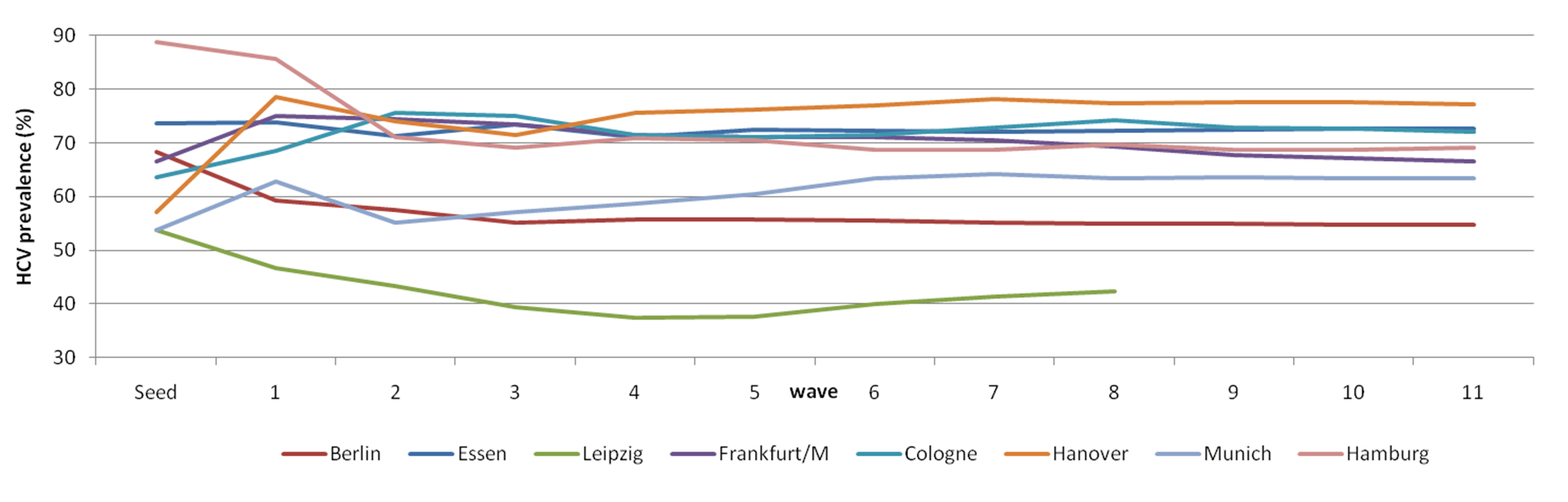

Supplement: Additional file 2: — HCV prevalence. (TIF 566 kb) [file 12889_2016_3545_MOESM2_ESM.tif]

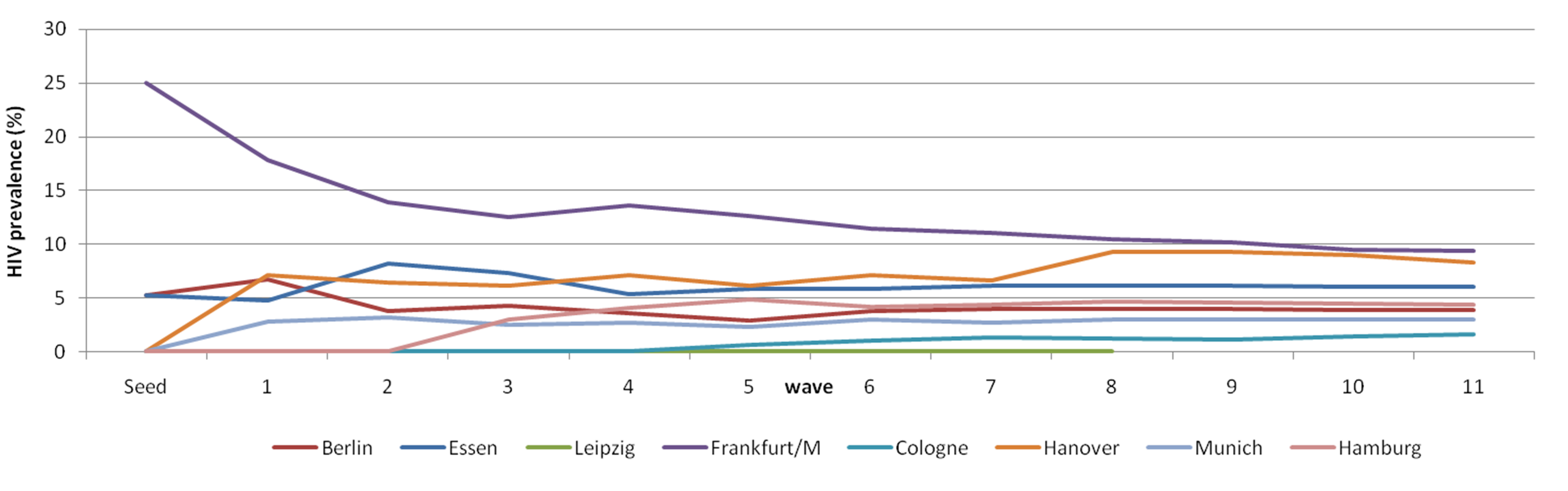

Supplement: Additional file 3: — HIV prevalence in all study cities except in Leipzig. (TIF 518 kb) [file 12889_2016_3545_MOESM3_ESM.tif]

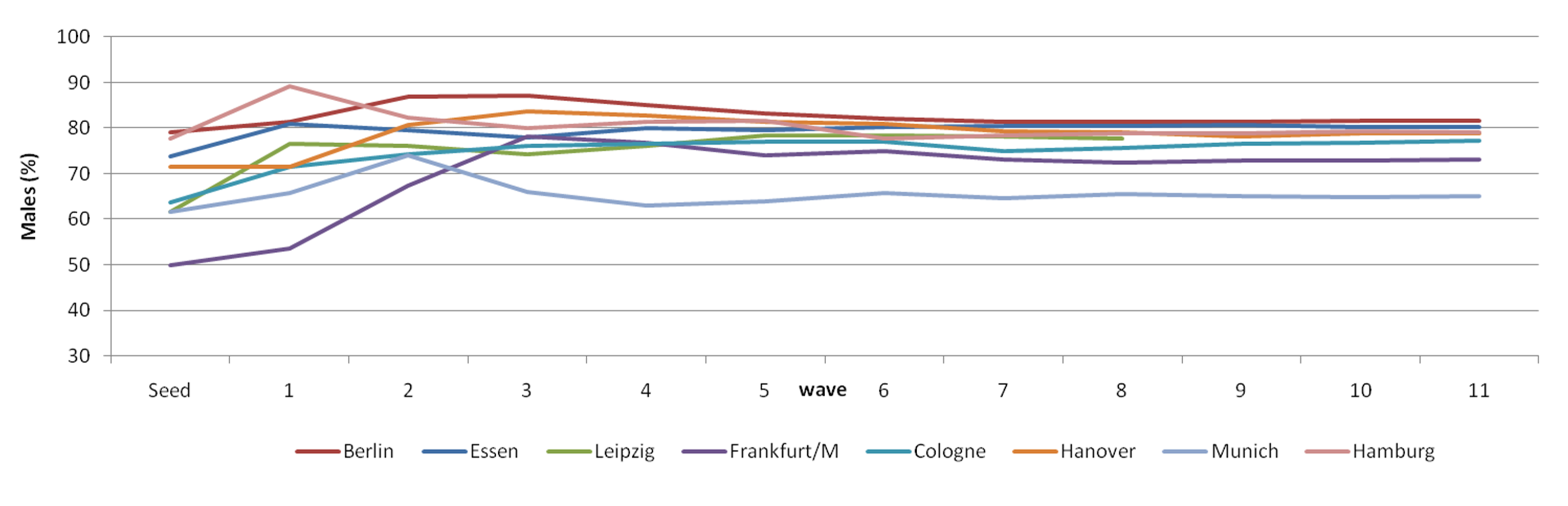

Supplement: Additional file 4: — Proportion of male participants. (TIF 535 kb) [file 12889_2016_3545_MOESM4_ESM.tif]

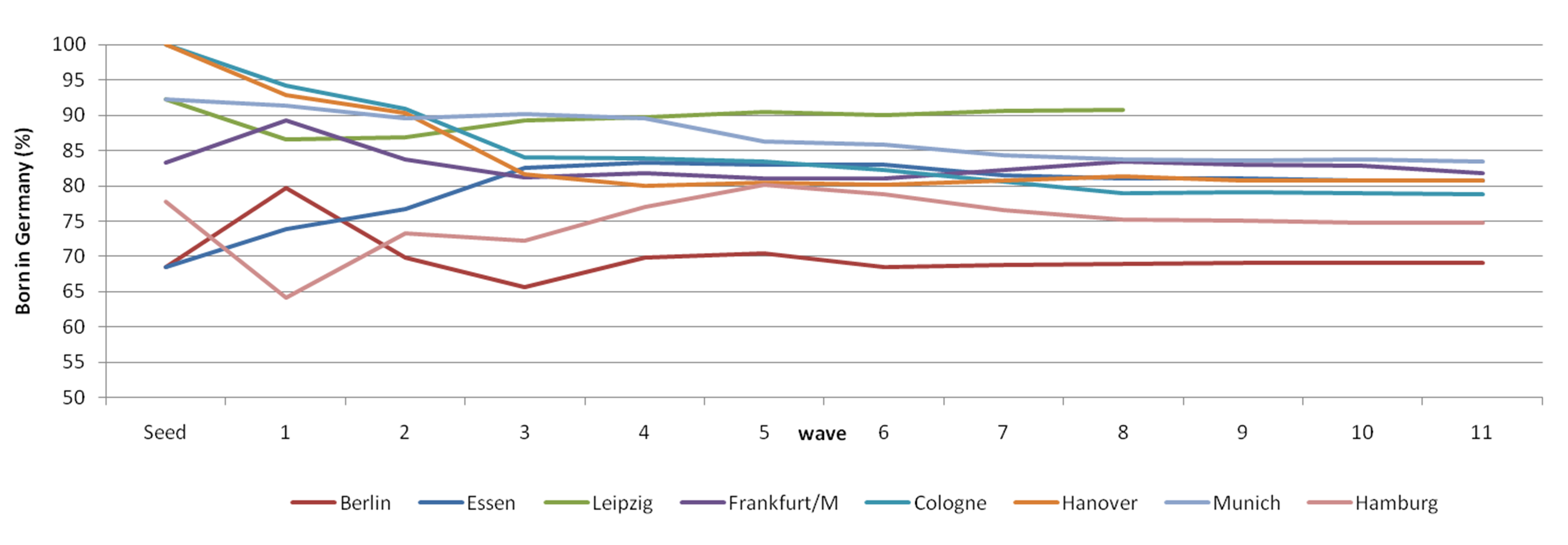

Supplement: Additional file 5: — Proportion of participants born in Germany. (TIF 628 kb) [file 12889_2016_3545_MOESM5_ESM.tif]

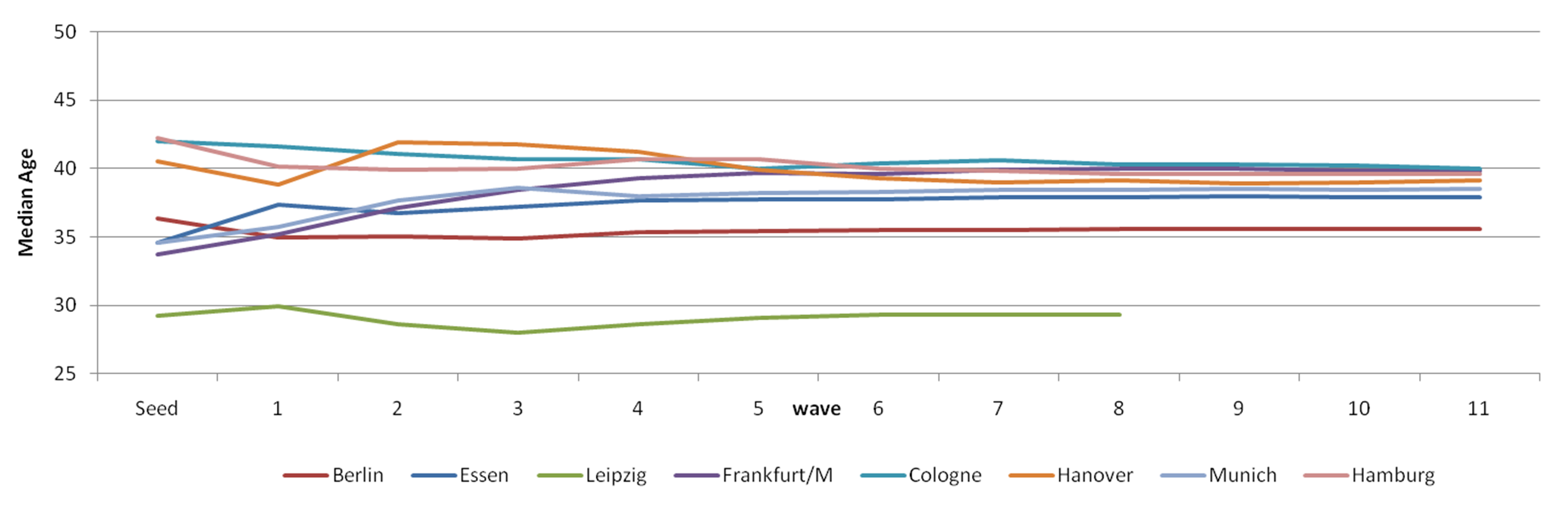

Supplement: Additional file 6: — Participants´ median age. (TIF 494 kb) [file 12889_2016_3545_MOESM6_ESM.tif]

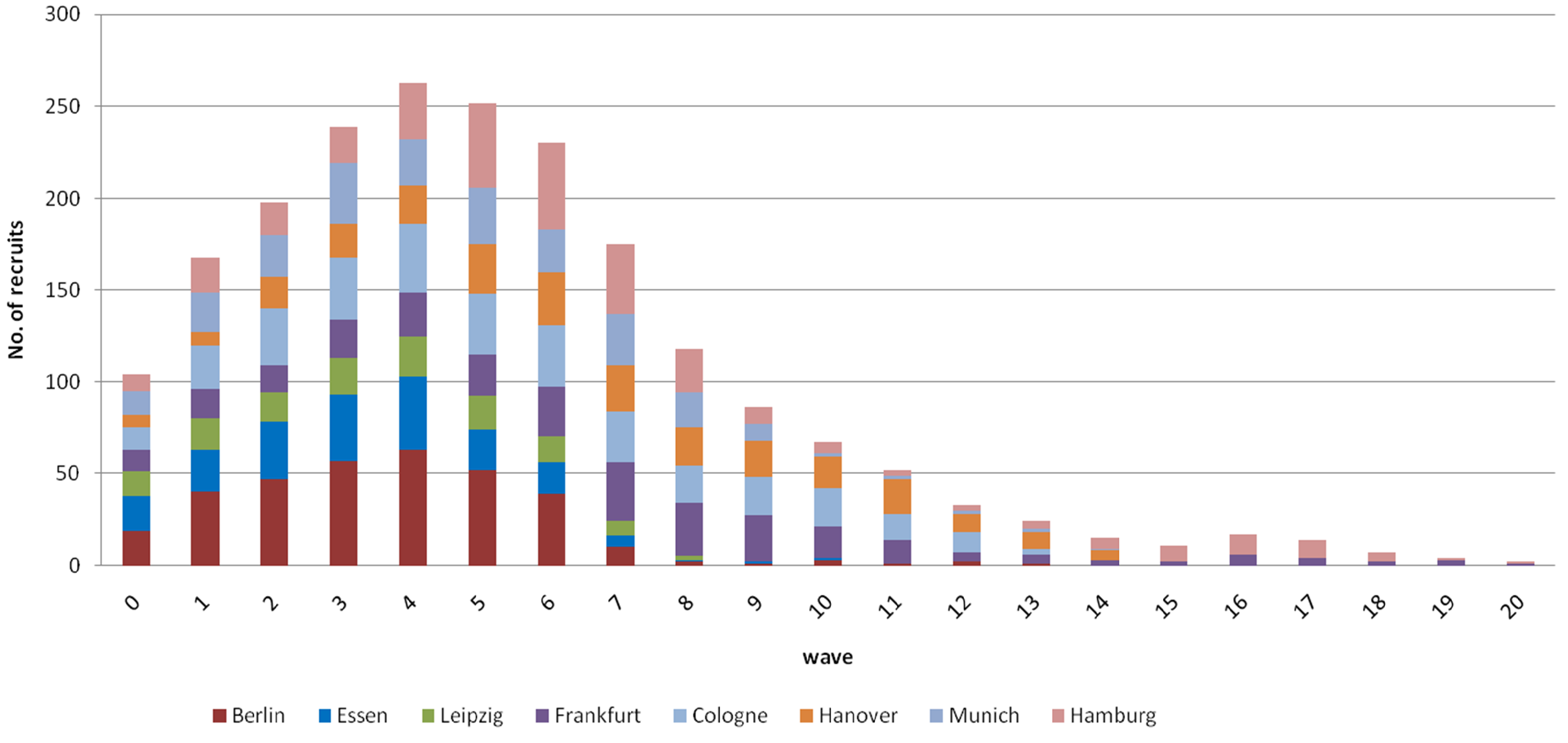

Supplement: Additional file 7: — Number of recruits per recruitment wave. (TIF 695 kb) [file 12889_2016_3545_MOESM7_ESM.tif]

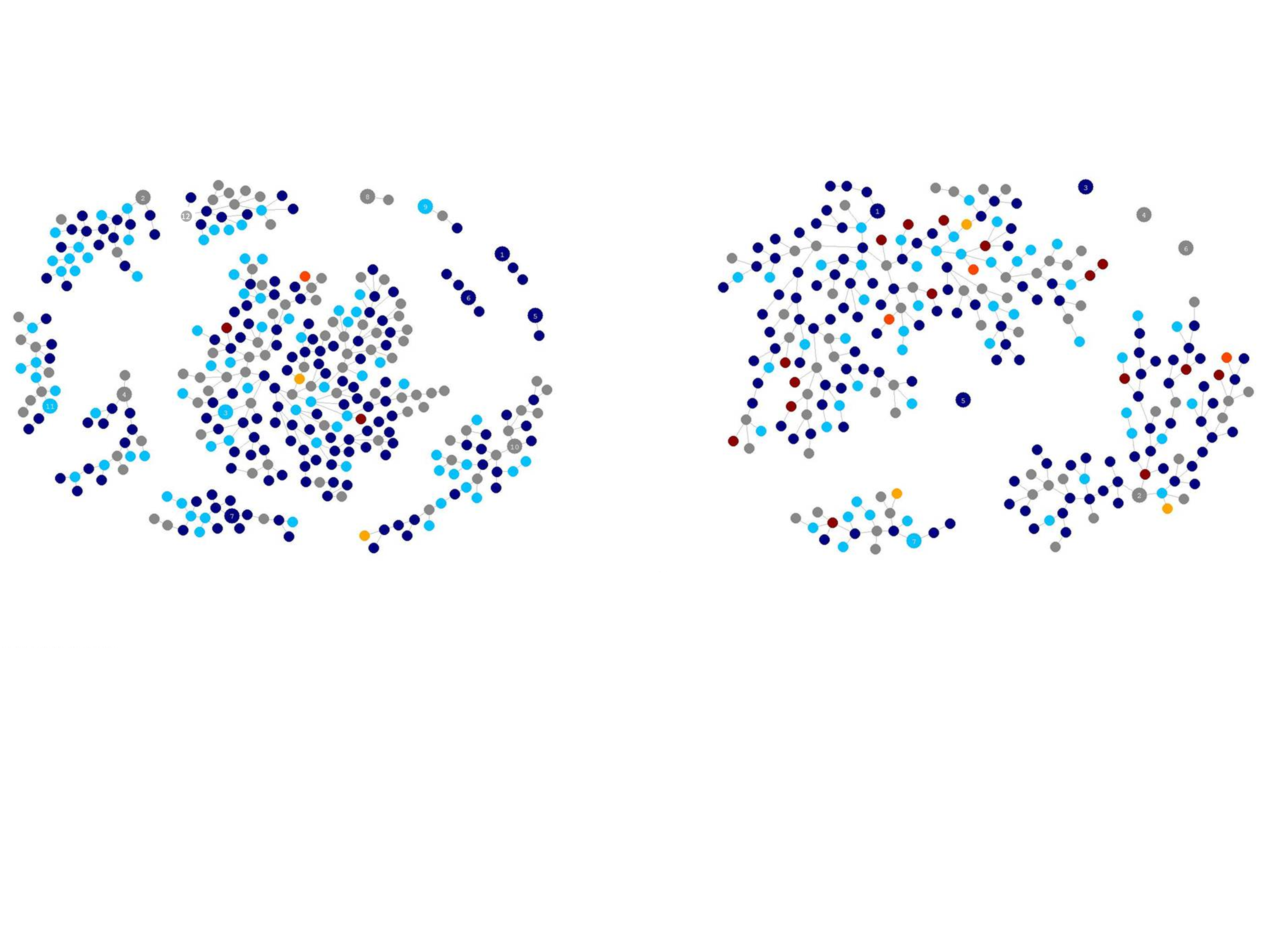

Supplement: Additional file 8: — Sample of Cologne (2013); n=322 (12 seeds) and sample of Hanover (2013); n=252 (7 seeds). (TIF 3197 kb) [file 12889_2016_3545_MOESM8_ESM.tif]

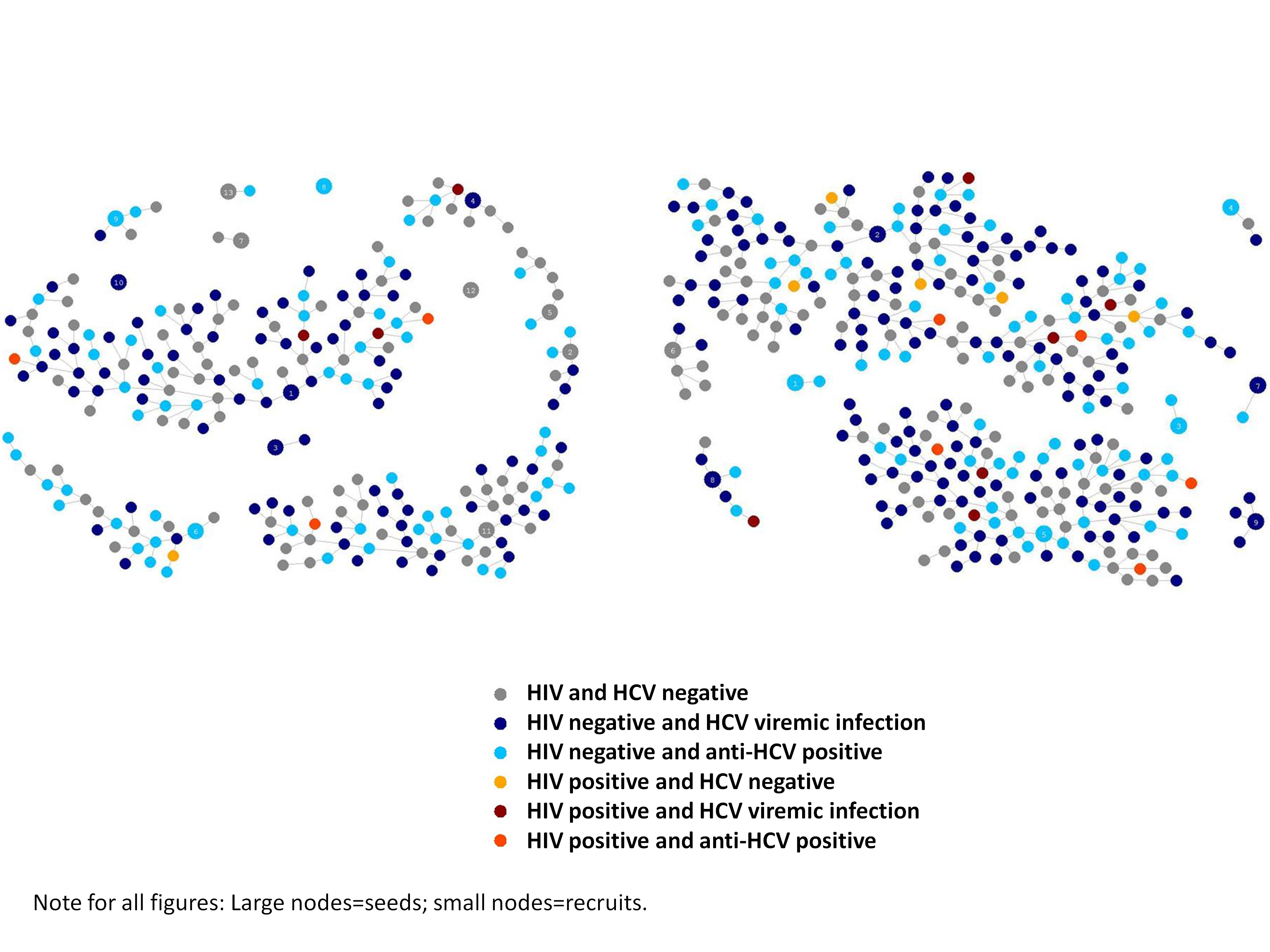

Supplement: Additional file 9: — Sample of Munich (2013); n=235 (13 seeds) and sample of Hamburg (2014); n=319 (9 seeds). (TIF 3828 kb) [file 12889_2016_3545_MOESM9_ESM.tif]

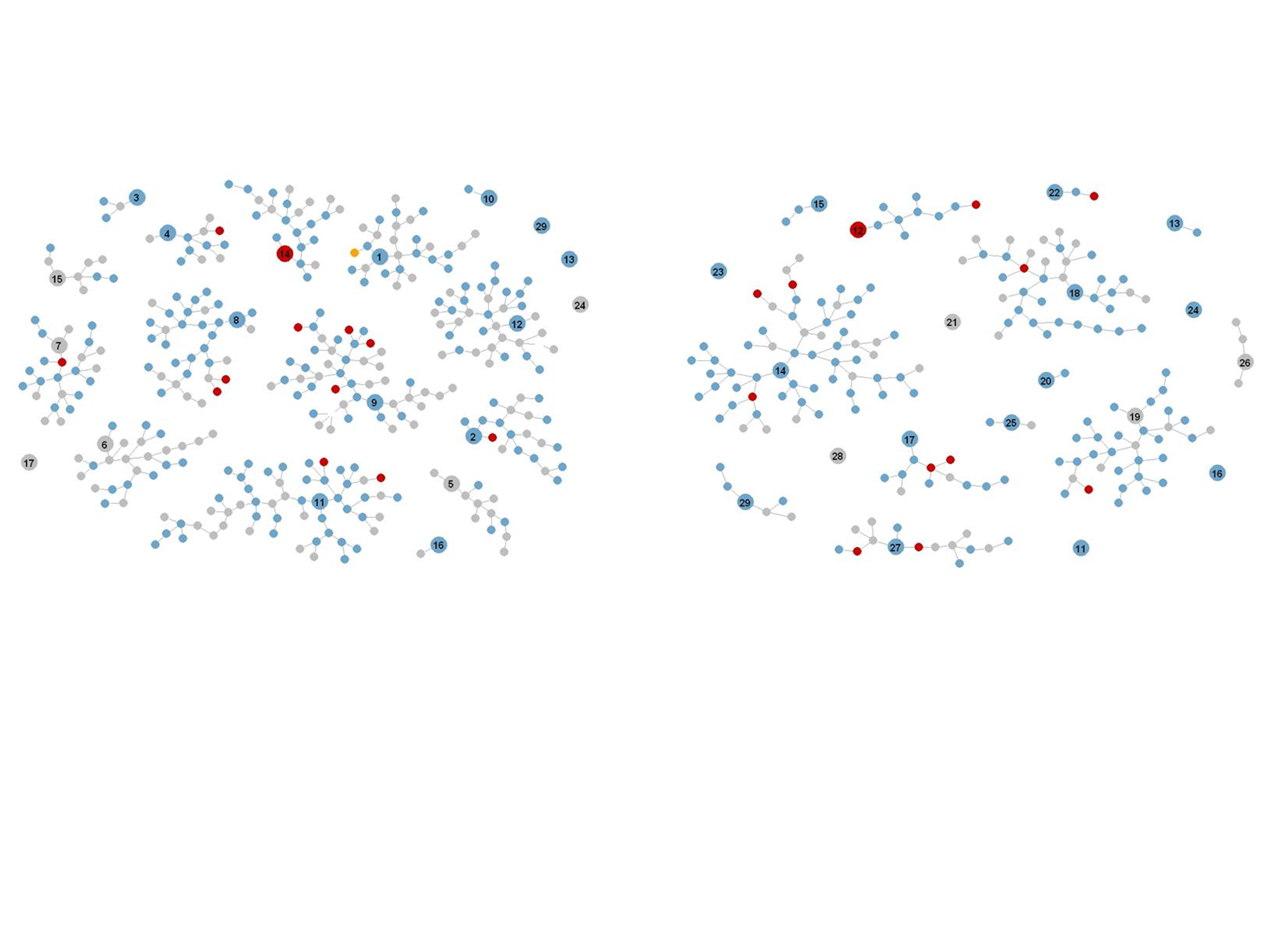

Supplement: Additional file 10: — Sample of Berlin (2011); n=337 (19 seeds) and sample of Essen (2011); n=197 (19 seeds). (TIF 2489 kb) [file 12889_2016_3545_MOESM10_ESM.tif]

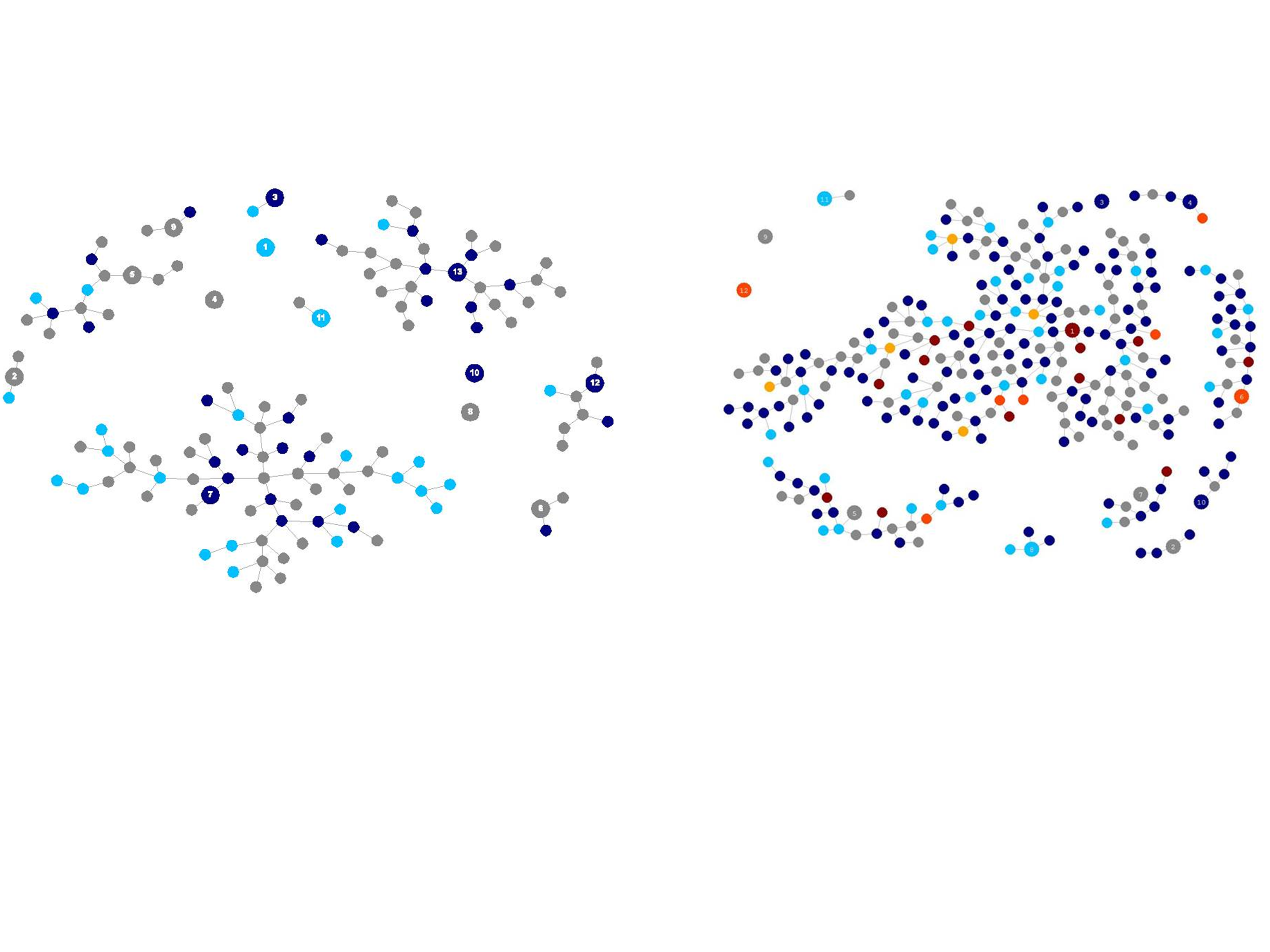

Supplement: Additional file 11: — Sample of Leipzig (2012); n=130 (13 seeds) and sample of Frankfurt (2013); n=285 (11 seeds). (TIF 2504 kb) [file 12889_2016_3545_MOESM11_ESM.tif]

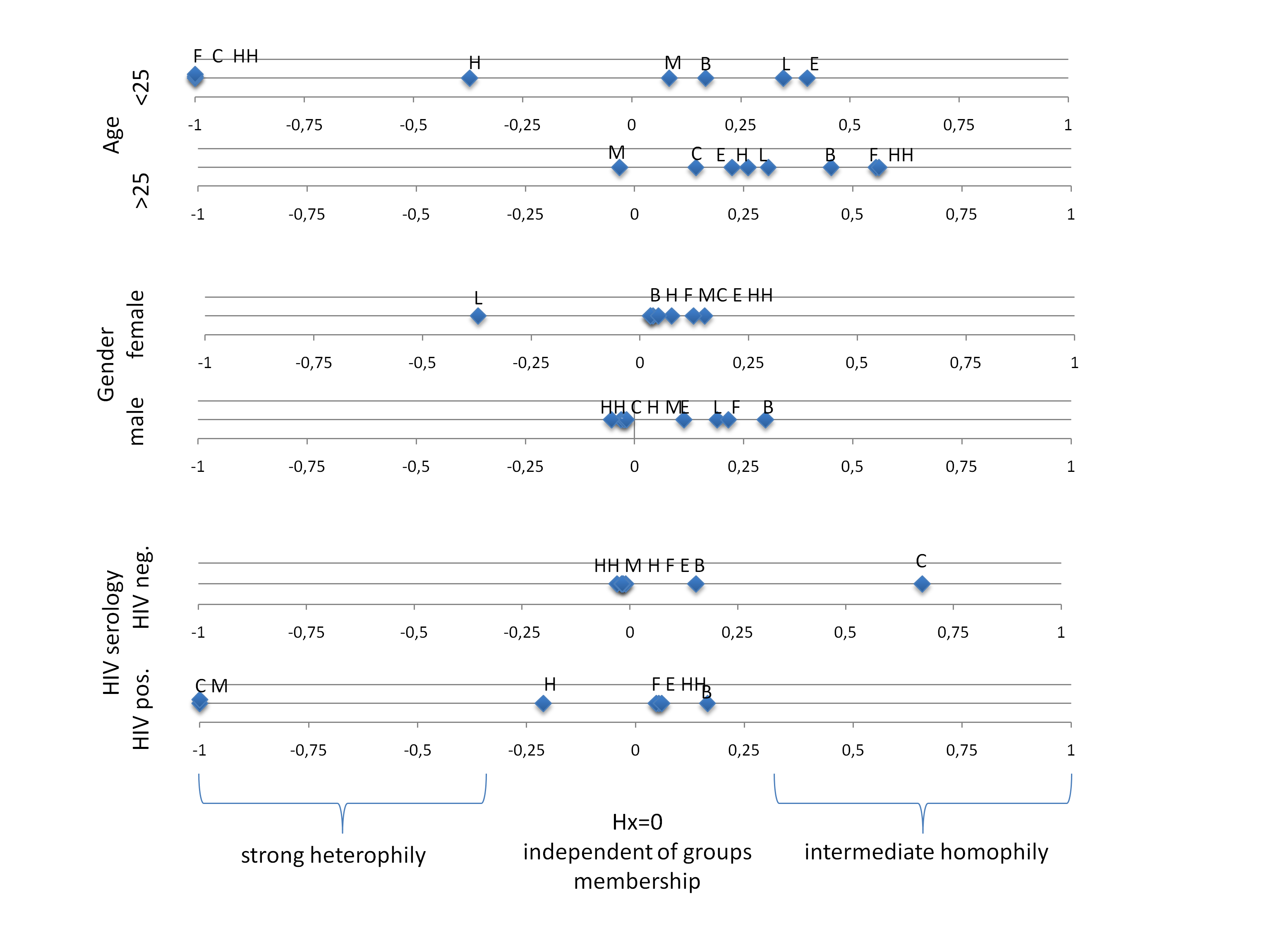

Supplement: Additional file 12: — Homophily (Hx) in eight city samples (age, gender, HIV seroprevalence). Cities: Berlin (B), Essen (E); Leipzig (L); Frankfurt (F); Cologne (C); Hanover (H); Munich (M); Hamburg (HH). The homophily Hx shows the tendency of individuals in a group having social bonds with other individuals similar to them. Hx = 0 means that the formation of social bonds is independent of group membership. Hx=1 mean no social bonds to outsiders exist. Hx= -1 means all social bonds are formed with people outside the group [44]. (TIF 690 kb) [file 12889_2016_3545_MOESM12_ESM.tif]
